# I-CONECT PROJECT: CAN SOCIAL INTERACTION IMPROVE COGNITIVE FUNCTIONS AMONG SOCIALLY ISOLATED OLDER ADULTS?

**DOI:** 10.1093/geroni/igz038.824

**Published:** 2019-11-08

**Authors:** Hiroko H Dodge, Karen Hooker, Toni C Antonucci

**Affiliations:** 1 University of Michigan, Ann Arbor, Michigan, United States; 2 Oregon State University, Corvallis, Oregon, United States

## Abstract

Epidemiological studies have demonstrated that larger social networks or more frequent social interactions may have protective effects against cognitive decline and the incidence of dementia. Therefore, increasing social interaction could be a promising intervention for improving cognitive well-being in socially isolated older adults. We have conducted multiple NIH-funded randomized controlled trials (RCT) over 10 years, examining whether conversational interactions through webcam and internet can improve cognitive functions and enhance cognitive reserve. In this symposium, we will introduce this series of behavioral intervention trials through 4 presentations. First, Dodge will provide background and results of their previous RCT where they showed efficacy of conversational intervention on domain-specific cognitive functions and introduce the ongoing larger project called I-CONECT (https://www.i-conect.org). Second, Lindsey will introduce technological innovations used in the I-CONECT project including development of user-friendly video-chat devices, recording of audio and video data and innovative recruitment efforts. Third, Asgari will share results on how speech utterance and characteristics collected through the project could distinguish those with mild cognitive impairment from those with normal cognition using machine learning modelling approaches. Finally, Cerino and his team will show results of the study which examined whether cognitive improvements through conversation-based intervention depend on an individual’s personality, laying the groundwork for a personalized intervention trial in the future. The symposium is of interest for those who study social isolation and its prevention, the link among cognition, social isolation and personality, as well as those who focus on technology as a tool for improving well-being of older adults.

